# First, Do No Harm: How Mental Health Service Responses Can Inadvertently Increase Suicidality Among Young People

**DOI:** 10.1177/13591045251383632

**Published:** 2025-09-25

**Authors:** Sarah Whitcombe-Dobbs

**Affiliations:** 1 2496 Faculty of Health, University of Canterbury| Te Whare Wananga o Waitaha, Christchurch, Aotearoa New Zealand

**Keywords:** developmental psychology, clinical psychology, child psychology, suicide, self-harm

## Abstract

Youth non-suicidal self-injury (NSSI) and suicidality have increased worldwide, yet funding is limited, and the workforce is stretched. This context of scarcity and risk influences service responses: thresholds for accessing services are raised, with increased gatekeeping. This commentary draws on behavioural and attachment theories to describe how reactive resource allocation can be harmful and inadvertently maintain distress. This occurs through unpredictably providing and then withdrawing access to mental health services. The mental health system itself can be seen as a participant in the therapeutic relationship, providing inconsistent and unpredictable responses to suicidal young people. Trauma- and attachment-related difficulties influence youth perceptions, and small interactions with services can maintain suicidality. When this pattern is recognised, it is possible to alter service responses to emulate interpersonal qualities that provide a sense of safety for those who seek help. For example, predictable responses, clear communication, validation throughout case progression, and consistent adherence to boundaries, all increase trust within the agency-client relationship.

## Plain Language Summary

Young people’s mental health is worsening worldwide. There is not enough help available. Self-harm and suicidal behaviours are common. Services have few resources, so make decisions about who can get help based on severity. Cases are closed and re-opened without consistency, and young people feel uncertain about whether they will get help or not. This can lead to young people feeling rejected by the service. Sometimes their suicidal behaviour increases because of this feeling. This is even more common in young people who have experienced trauma in their lives before engaging with the service. This pattern between the service and the young person can be harmful, even though the service is trying to help. Instead, services should try to behave like a consistent and caring parent, to help young people to feel more secure. For example, services can make sure they communicate with the young person in a predictable manner. They can also show the young person they care, by telling them that their feelings are valid and taking their concerns seriously. Services can also make sure they follow their own rules, such as keeping information confidential. When services do these kinds of things, then young people and their families are more likely to trust the service and feel supported. This can lead to young people being safer overall.

## Introduction

### Context: The Youth Mental Health Crisis

Over the past two decades, there has been a sharp increase in reported psychological distress among youth, and in particular an increase in suicidality and non-suicidal self-injury (NSSI) (Bertuccio et al., 2024; Piao et al., 2022; Young et al., 2025). This mental health crisis has caused consternation among researchers, professionals, and the public, yet no significant improvements or trend reversals have occurred (Bradley, 2023; Fleming et al., 2022; Menzies et al., 2020). Many sociocultural factors contribute to mental health outcomes (e.g. Hashemi et al., 2021; Kisely et al., 2021; Telfar et al., 2023), and there is a simple mismatch between mental health needs among youth and the capacity of available services. However, when youth with severe and complex mental health problems do access mental health services, these can be ineffective and families are left feeling frightened and frustrated (Every-Palmer et al., 2022; Gilmour et al., 2019; Taylor et al., 2018). Youth who are high service-users, and who present with chronic suicidality, are difficult to treat (Burns et al., 2008; McCutcheon et al., 2007; Thabrew et al., 2018).

Previous literature has explored the dynamics surrounding youth NSSI and suicidality with some depth (e.g. Grandclerc et al., 2016; Sadath et al., 2024; Simes et al., 2022). Acheson and Papadima (2023) frame the recent escalation in terms of adolescent identity development and relational need. This is situated within a current ecological context that increases distress, while also reinforcing the expression of the same through diagnostic language and suicidal discourse. Within services, there is a shift away from meaning-making and relational foci towards risk management and the supply of strategies via short-term treatments for discrete disorders. This type of practice risks an escalation pathway towards adult-oriented case conceptualisation, such as personality disorders and the anticipation of long-term relational and emotional instability (Acheson & Papadima, 2023).

A case example allows for an in-depth exploration of youth experiences and behaviour patterns, in a specific system of care. Aotearoa New Zealand (A/NZ) has a nationwide health system that provides community-based and inpatient mental health services to children and adolescents. Although there are some regional variations, Te Whatu Ora (The Ministry of Health) oversees the universal free public service, pathways for accessing this are similar across the whole country. Sutcliffe and colleagues (2024) conducted a cluster analysis of youth self-report data in A/NZ, which yielded results indicating alarming levels of psychological distress. The two most severe clusters comprised 22% of the sample, with the final group of 6.4% having high internalising scores, NSSI, and a reported previous suicide attempt (Sutcliffe et al., 2022, 2024). These data suggest that about one in five A/NZ adolescents needs assessment and psychological support, with about one in twenty needing ongoing monitoring and treatment.

Mental health presentations to hospitals have drastically increased over the past decade (Young et al., 2025), and specialist out- and in-patient services in Aotearoa/NZ do not currently have capacity to meet this need (Haycock, 2023). This leaves ineligible youth relying largely on psychopharmacological treatment through their GP, or private practitioners if they are fortunate. Recommendations for mental health apps or text and crisis numbers for support abound, but these do not provide individualised treatment (Healthify He Puna Waiora, 2024). There is a high prevalence of complex and severe presentations among youth referred to Child and Adolescent Mental Health Services (CAMHS). By the time they are seen in person, youth may already have attempted suicide, and families are very frightened for their safety (NZ Government, 2018).

There is a mismatch between the level of need and the capacity of the available services (Every-Palmer et al., 2022; NZ Government, 2018). This results in inadvertently perverse service responses that increase youth mental distress. This commentary therefore aims to do the following, using A/NZ as a case example: (1) describe how behavioural and attachment theories underpin youth mental health presentations, (2) describe how young people’s interactions with mental health services are influenced by their relational perceptions, and (3) describe key clinical implications.

### Behavioural Theory – Its Relevance to Youth Mental Health Presentations

The intermittent schedule of reinforcement is a powerful driver for human behaviour. In a youth mental health setting, intermittent access to treatment can create a higher rate of bids for services. In behavioural terms, this withdrawal of service provision constitutes a negative punishment, or the removal of something desirable. This makes it less likely that the preceding behaviour, the reporting of symptom improvement, will re-occur. This can also be framed as positive reinforcement, whereby the addition of services increases the likelihood that the help-seeking behaviour will re-occur. Mental health practitioners are aware of this, and youth suicidality can sometimes be seen by them and other young people as ‘attention-seeking’ or ‘manipulative’, with service responses escalated only if it is seen as serious and genuine (Crouch & Wright, 2004; Dixon-Ward & Chan, 2022). Sometimes, professionals refuse to treat in an attempt to avoid this dynamic – with the belief that ‘you have to be cruel to be kind’ (MacDonald et al., 2021; Williams, 1998). Phrases are used, such as saying a young person ‘has an intent to die’ to differentiate those who are deemed in need of treatment from those whose suicide attempts are deemed non-serious (and therefore may be safely ignored) (Andover et al., 2012). It is inaccurate to view NSSI and suicide attempts as non-serious – risk assessment is an imprecise science, and NSSI and former suicide attempts are a significant predictor of completed suicide (Grandclerc et al., 2016).

### Attachment Theory – Its Relevance to Youth Mental Health Presentations

Attachment develops in infancy, with caregiving responses over time shaping a person’s expectations for comfort and safety. Describing the relationship between a person and those close to them, attachment is observable and can be classified, and has implications for child development (Sroufe et al., 1999). Anxious attachment is characterised by heightened emotional expression, a strong need for closeness, worries about relationships, fear of rejection, and a ‘self-amplifying cycle of distress’ (Pearse et al., 2020). Many youth with severe mental health needs are unable to self-regulate and self-soothe, and can engage in ‘resist-refuse’ or ‘push-pull’ behaviours when professional validation is offered (Swee et al., 2020). Some of these may seem developmentally typical for the adolescent period but are amplified when clients are psychologically distressed. For mental health professionals under stress themselves, this can be confusing and frustrating (Leddie et al., 2022), and sometimes help can be withdrawn as a result.

### Typical Service Responses

Services intend to be ‘minimally sufficient’ to avoid unnecessary service provision. Staff also consider whether a client’s referral warrants a service when compared to others’ needs. Intake workers therefore create formal or informal thresholds for access to services, and triaging work involves determining the severity of the client’s presentation (MacDonald et al., 2021). These service thresholds vary considerably, and can depend on service capacity at the time of referral (Barbour et al., 2002).

**Figure 1:**
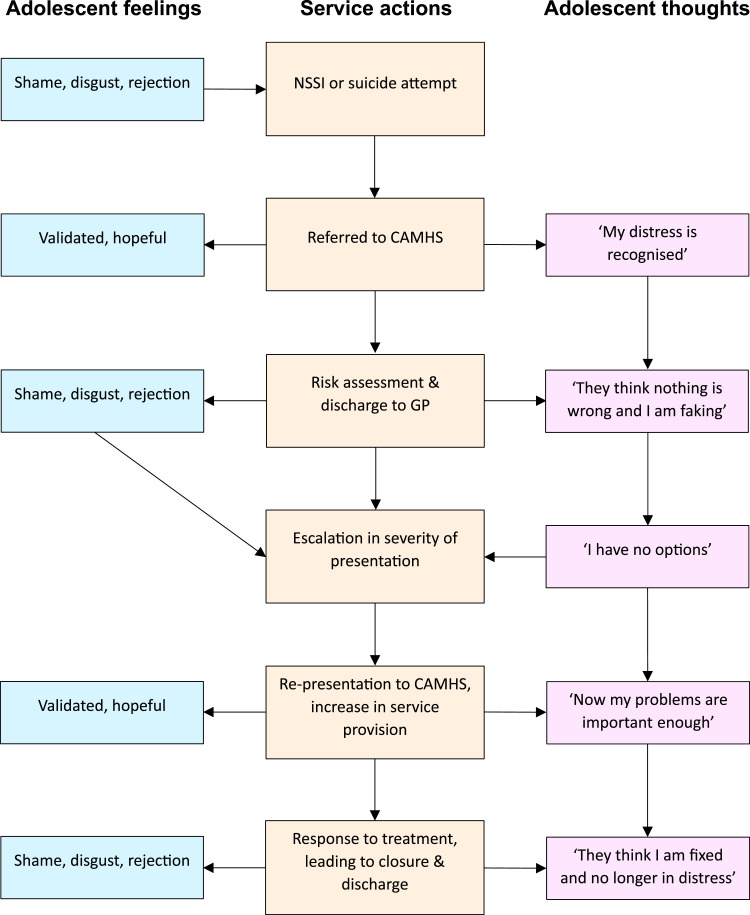
Hypothetical Adolescent Thoughts and Feelings Alongside CAMHS Case Progression

The following describes a typical treatment pathway in A/NZ (see Figure 1) (Miller & James, 2025). Following referral, professionals undertake risk assessment at an initial appointment, and often the outcome is that the referral is subsequently discharged to their GP. Sometimes a safety plan is made and shared with the young person and their family, and sometimes not. Symptoms may initially reduce, but a re-escalation of distress often occurs, resulting in several re-presentations to CAMHS or emergency departments (Young et al., 2025). Ongoing services may then be provided and the young person and their family may receive further assessment, case management, and perhaps short-term therapy (Thabrew et al., 2018). This often feels like a relief for the young person and their family: professionals are providing help and support. Following an improvement, the case is then discharged again, leading to another cycle of escalation and re-presentation. This is one example, yet the principle is the same – there is a provision and withdrawal of services that rests on the agency’s perception of the young person’s level of risk and need.

## Perceptions of Typical Service Responses

Feelings of shame, self-disgust and rejection often accompany instances of NSSI or suicide attempts (Chu et al., 2017; Sadath et al., 2024; Schienle et al., 2020). Adolescents receiving treatment in A/NZ for suicidality are more likely than other CAMHS clients to have more complex backgrounds and experiences, including sexual abuse, substance use, and a range of family psychosocial stressors (Fortune & Lambie, 2005). Young people feel lonely and do not want to be a burden (Buitron et al., 2016). When receiving a service, recognition by others can lead to feeling validated and hopeful. Health professionals are empathetic and see the situation as important to address, and this is felt as reassuring – although also sometimes frightening and uncomfortable (Marzetti et al., 2023).

Clients’ perceptions of health professionals are shaped by prior experiences of asking for help and their own pre-existing attachment representations. When discharge inevitably occurs, young people may interpret this as professionals thinking that there is nothing wrong, or that they were faking or exaggerating their distress (Dixon-Ward & Chan, 2022). Youth may feel as though they cannot express their distress without suicidality – the ‘emergency siren’ that quickly elicits reassurance. They may feel personally betrayed or rejected, resulting in a deep sense of shame and anger (Sadath et al., 2024). Sometimes, this intense emotion can lead to a further escalation in externalising or internalising problems, which in turn ‘force’ services to provide more help in response to crisis. Throughout this cycle of service response and discharge, there are many micro-interactions that alternate between perceived validation and rejection, serving to create relational uncertainty and increase anxiety in the client (see Figure 1). This type of suicidal cycle in young people is sometimes framed as attention-seeking, or an emerging personality disorder (Moselli et al., 2023). Indeed, some clients can be unpredictable, with relational actions echoing the response of an infant with a disorganised attachment style (Zortea et al., 2021). For example, affect dysregulation occurs along with hostile, dissociative, or avoidant responses (Černis et al., 2019). Health professionals can feel confused and frustrated when offered help is rejected or ignored, leading to their own frustration – which is also sometimes expressed towards the young person (Gvion et al., 2021).

Young people are aware that when assessing referrals, professionals ask about suicidality to determine whether they should be helped. Marzetti and colleagues (2023) explored A/NZ youth experiences of suicidality, finding that some participants felt it was necessary to disclose suicidality and NSSI in order to get help. Participant Yasmin said, *I had to be like I’m going to kill myself if you don’t refer me [for mental health support]. Like I had to say that to her [Yasmin’s GP] more than once…because she had already been dismissive, but I felt this is the only way…* (p. 509, Marzetti et al., 2023). Another study of A/NZ young people who used crisis helplines captured the uncertainty while trying to access help in the midst of distress. One participant expressed this, saying *Uhm, it varies all the time and it’s quite hard to experience that because you’re in distress and you don’t know if you’re going to get a response or yeah, just you don’t know if you’re going to get a response* (Stace, 2024, p. 87). Media stories on the youth mental health crisis in A/NZ have also highlighted the perception from young people that they need to attempt suicide, or at least be in crisis, in order to be listened to (e.g. Bradley, 2023; Cooke, 2021).

A meta-ethnography of qualitative studies, examining youth experiences of mental health services, summarised the findings of four studies (Gilmour et al., 2019). These highlighted the difficulty in finding and then accessing mental health services, feelings of shame and lack of trust in professionals, and the importance of face-to-face engagement, connection, and honest communication. Primarily, suicidal young people wanted to be heard, but were alert to perceived slights: *I had very high standards for people. . .if they looked down on me or said something that made me mad, just something a bit off beat, it would completely ruin it. I’d want to leave…* (Murray & Wright, 2006, p. 161). There can be an interpersonal function when seeking help for suicidality, that includes the need to communicate to others the extremity of their felt distress (Steggals et al., 2020). Some health professionals recognise this cycle of distress and understand that it is largely outside a young person’s conscious control. Yet when discharge is done well following effective treatment, a sense of invalidation is less likely. This is because the decision has been reached through collaborative decision-making and a shared understanding of the young person’s progress via measurement-based care. This latter approach requires considerable skill, but in turn amplifies clients’ agency, rather than undermining it (Acheson & Papadima, 2023).

Mental health services themselves thus become a participant within the therapeutic relationship. Some formulation models explicitly describe patterns between families and agencies (e.g. Connor & Fisher, 1997), but services themselves do not evaluate their own effectiveness with these in mind. If the agency was a parent, they would be alternating warm responsiveness with dismissive behaviour, eliciting feelings of anxiety and uncertainty for those on the receiving end (Jones et al., 2015; Marzetti et al., 2023). For young people who struggle to take others’ perspectives and to self-regulate, the uncertainty and perceived invalidation elicit attachment-related distress.

Services are operating within a pressured system, and prioritise based on the highest perceived need (Haycock, 2023). Yet families are seeking to ‘create a trusting and respectful relationship between a child or young person, their family, and a therapeutic team’ (NZ Government, 2018, p. 49). The analogy of the parent-child relationship reveals some simple approaches that are within the control of the service, which relate to increasing security and trust within relationships. These include predictability, emotional availability, sensitivity and attunement, consistent boundaries, and consistency across settings (Morris et al., 2017).

### Key Implications and Recommendations for Clinical Practice

Despite the strains on the mental health system, practice changes can be implemented within systems and by individual practitioners that may alter the patterns and experiences described in previous sections. Some general guidelines and recommendations are outlined below, intended to aid professionals who want to avoid this interactional process. These may also help inform health system managers in reviewing and improving system effectiveness. Key themes include increasing agency predictability, providing sensitive and flexible care, having clear boundaries, and improving the consistency of practice across different parts of the system.

Improve service predictability wherever possible, by communicating in a manner that is easily understood and checking for this understanding before moving on (Taylor et al., 2018). Slowing down and allowing time to digest information aids comprehension. When in crisis, it can be hard to remember what a professional has said, so written outcomes are valuable, or even allowing families to voice-record summaries and next steps on their phones so they can listen again later. Young people and families often say they have understood something, or agree to a plan out of a desire to please professionals (Coyne et al., 2015). In A/NZ this is particularly true for Māori, Pacific people, people whose English is a second language, or who come from cultures where health professionals hold high status (NZ Government, 2018). Even though this takes a little more time, it can increase families’ confidence in their own understanding, reducing the likelihood of unnecessary re-presentations.

Cultivate awareness that young people may interpret discharge as a personal rejection or an invalidation of their distress (Taylor et al., 2018; Wasson Simpson et al., 2022). Directly address this during discussions with the young person and their family and acknowledge that case closure does not mean that all problems are resolved. Minimising the remaining issues, in an attempt to justify the decision, can feel dismissive (Idenfors et al., 2015). Allow young people to express their feelings in response to discharge. Feelings of rejection may be prevented, especially when this follows ongoing discussions with the young person regarding their progress in treatment. It also provides the opportunity for a more detailed intervention and management plan to be created collaboratively, increasing youth safety (Simes et al., 2022). Reminding young people that a re-referral to mental health services is an option can reduce the feeling of rejection – and they should receive specific written information on how to do this (Idenfors et al., 2015). This supports youth autonomy and provides the sense of the service as a ‘safe haven and secure base’, encouraging a type of attachment between the client and the service (Pitama et al., 2007).

Undertake risk assessment and other high-stakes engagements sensitively, considering the young person’s emotional state at the time and their perceptions of your motivation. Repeated screening for suicidality does not increase risk (Hom et al., 2018), but it can send the message that rapport-building only occurs with the goal of gaining an accurate risk assessment (Taylor et al., 2018). Do not finish the engagement immediately following the direct questioning, instead use opening and closing processes in a balanced manner (Lacey et al., 2011). Sensitivity can be manifested through noticing individual young people’s characteristics, remembering these, and giving genuine attention with curiosity and humour (McCutcheon et al., 2007). Give extra time to the engagement where possible, without an agenda of completing processes. In A/NZ, young people see a relaxed professional connection, and sharing of backgrounds, as particularly important (Stubbing & Gibson, 2022).

Consistent boundaries are recommended for practitioners and services, as well as with young people accessing the service (Taylor et al., 2018). These are challenging within a healthcare setting, often due to factors outside practitioners’ control. This would look like services adhering to stated timeframes, and being clear about reasons and alternative options when referrals are denied (NZ Government, 2018; Taylor et al., 2018). Confidentiality must be upheld when promised, even from other professionals within the service. At the same time, when information needs to be shared this should be done promptly and following correct processes. Practitioners working with a new young person should read any previous plans or reports, and contact other services when requested to do so (O'Reilly et al., 2013). In A/NZ, ensuring the GP receives instructions on aftercare, cc’d to the young person themselves, would demonstrate this consistency of care. These types of actions can prevent family frustration with contradictory professional advice, and having to share personal information repeatedly – and can increase trust (Gilmour et al., 2019; McCutcheon et al., 2007; Taylor et al., 2018).

## Conclusion

Systemic responses to youth suicidality can be lifesaving, but they can also inadvertently increase distress and therefore risk for further harm. Behavioural and attachment-related factors can inadvertently combine to drive suicidality, even while services are being provided. Given this pattern, CAMHS services and mental health practitioners should address these directly, alongside implementing principles of effective practice that have been well covered in other literature (e.g. Eapen et al., 2022; McGorry et al., 2018; Stubbing & Gibson, 2021). In implementing these, conceptualising the service-young person relationship as analogous to the parent-child relationship will lead to more compassionate and effective treatment. Ultimately, services must critically examine their own response patterns, and change these, to improve safety for youth with chronic suicidality.

## References

[bibr1-13591045251383632] Acheson R. Papadima M. (2023). The search for identity: Working therapeutically with adolescents in crisis. Journal of Child Psychotherapy, 49(1), 95–119. 10.1080/0075417X.2022.2160478

[bibr2-13591045251383632] Andover M. S. Morris B. W. Wren A. Bruzzese M. E. (2012). The co-occurrence of non-suicidal self-injury and attempted suicide among adolescents: Distinguishing risk factors and psychosocial correlates. Child and Adolescent Psychiatry and Mental Health, 6(1), 11. 10.1186/1753-2000-6-11 22463065 PMC3379960

[bibr3-13591045251383632] Barbour R. S. Stanley N. Penhale B. Holden S. (2002). Assessing risk: Professional perspectives on work involving mental health and child care services. Journal of Interprofessional Care, 16(4), 323–334. 10.1080/1356182021000008256 12487841

[bibr4-13591045251383632] Bertuccio P. Amerio A. Grande E. La Vecchia C. Costanza A. Aguglia A. Berardelli I. Serafini G. Amore M. Pompili M. Odone A. (2024). Global trends in youth suicide from 1990 to 2020: An analysis of data from the WHO mortality database. eClinicalMedicine, 70, 102506. 10.1016/j.eclinm.2024.102506 38440131 PMC10911948

[bibr5-13591045251383632] Bradley A. (2023). The ‘rolling crisis’ in youth mental health that leaves thousands waiting weeks for help. https://www.rnz.co.nz/programmes/in-depth-special-projects/story/2018919377/the-rolling-crisis-in-youth-mental-health-that-leaves-thousands-waiting-weeks-for-help

[bibr6-13591045251383632] Buitron V. Hill R. M. Pettit J. W. Green K. L. Hatkevich C. Sharp C. (2016). Interpersonal stress and suicidal ideation in adolescence: An indirect association through perceived burdensomeness toward others. Journal of Affective Disorders, 190, 143–149. 10.1016/j.jad.2015.09.077 26519633

[bibr7-13591045251383632] Burns C. D. Cortell R. Wagner B. M. (2008). Treatment compliance in adolescents after attempted suicide: A 2-year follow-up study. Journal of the American Academy of Child and Adolescent Psychiatry, 47(8), 948–957. 10.1097/CHI.Ob013e3181799e84 18596554 PMC2637135

[bibr8-13591045251383632] Černis E. Chan C. Cooper M. (2019). What is the relationship between dissociation and self-harming behaviour in adolescents? Clinical Psychology & Psychotherapy, 26(3), 328–338. 10.1002/cpp.2354 30690804

[bibr9-13591045251383632] Chu C. Buchman-Schmitt J. M. Stanley I. H. Hom M. A. Tucker R. P. Hagan C. R. Rogers M. L. Podlogar M. C. Chiurliza B. Ringer F. B. Michaels M. S. Patros C. H. G. Joiner T. E. (2017). The interpersonal theory of suicide: A systematic review and meta-analysis of a decade of cross-national research. Psychological Bulletin, 143(12), 1313–1345. 10.1037/bul0000123 29072480 PMC5730496

[bibr10-13591045251383632] Connor D. F. Fisher S. G. (1997). An interactional model of child and adolescent mental health clinical case formulation. Clinical Child Psychology and Psychiatry, 2(3), 353–368. 10.1177/1359104597023004

[bibr11-13591045251383632] Cooke M. (2021). Teen shares experience of mental health system: 'It feels like no one is listening. https://www.rnz.co.nz/news/national/457865/teen-shares-experience-of-mental-health-system-it-feels-like-no-one-is-listening

[bibr12-13591045251383632] Coyne I. McNamara N. Healy M. Gower C. Sarkar M. McNicholas F. (2015). Adolescents' and parents' views of child and adolescent mental health services (CAMHS) in Ireland. Journal of Psychiatric and Mental Health Nursing, 22(8), 561–569. 10.1111/jpm.12215 25977175

[bibr13-13591045251383632] Crouch W. Wright J. (2004). Deliberate self-harm at an adolescent unit: A qualitative investigation. Clinical Child Psychology and Psychiatry, 9(2), 185–204. 10.1177/1359104504041918

[bibr14-13591045251383632] Dixon-Ward K. C. Chan S. W. Y. (2022). ‘Faking it’: Exploring adolescent perceptions of depression (in)authenticity and ‘attention seeking. The British Journal of Clinical Psychology, 61(2), 177–196. 10.1111/bjc.12339 34716598

[bibr15-13591045251383632] Eapen V. Stylianakis A. Scott E. Milroy H. Bowden M. Haslam R. Stathis S. (2022). Stemming the tide of mental health problems in young people: Challenges and potential solutions. The Australian and New Zealand Journal of Psychiatry, 57(4), 482–488. 10.1177/00048674221136037 36377648

[bibr16-13591045251383632] Every-Palmer S. Grant M. L. Thabrew H. (2022). Young people don’t tend to ask for help more than once: Child and adolescent psychiatrists’ views on ailing mental health services for young New Zealanders. Australasian Psychiatry: Bulletin of Royal Australian and New Zealand College of Psychiatrists, 30(6), 684–688. 10.1177/10398562221115624 35918633

[bibr17-13591045251383632] Fleming T. Ball J. Bavin L. Rivera-Rodriguez C. Peiris-John R. Crengle S. Sutcliffe K. Lewycka S. Archer D. Clark T. C. (2022). Mixed progress in adolescent health and wellbeing in Aotearoa New Zealand 2001–2019: A population overview from the Youth2000 survey series. Journal of the Royal Society of New Zealand, 52(4), 426–449. 10.1080/03036758.2022.2072349 39440321 PMC11485765

[bibr18-13591045251383632] Fortune S. Lambie I. (2005). Suicide behaviour in a clinical sample of children and adolescents in New Zealand. New Zealand Journal of Psychology, 34(3), 164–170. https://www.psychology.org.nz/journal-archive/NZJP-Vol343-2005-4-Fortune.pdf

[bibr19-13591045251383632] Gilmour L. Ring N. Maxwell M. (2019). Review: The views and experiences of suicidal children and young people of mental health support services: A meta-ethnography. Child and Adolescent Mental Health, 24(3), 217–229. 10.1111/camh.12328 32677214

[bibr20-13591045251383632] Grandclerc S. De Labrouhe D. Spodenkiewicz M. Lachal J. Moro M.-R. (2016). Relations between nonsuicidal self-injury and suicidal behavior in adolescence: A systematic review. PLoS One, 11(4), Article e0153760. 10.1371/journal.pone.0153760 27089157 PMC4835048

[bibr21-13591045251383632] Gvion Y. Rozett H. Stern T. (2021). Will you agree to treat a suicidal adolescent? A comparative study among mental health professionals. European Child & Adolescent Psychiatry, 30(4), 671–680. 10.1007/s00787-020-01581-w 32661613

[bibr22-13591045251383632] Hashemi L. Fanslow J. Gulliver P. McIntosh T. (2021). Exploring the health burden of cumulative and specific adverse childhood experiences in New Zealand: Results from a population-based study. Child Abuse & Neglect, 122, 105372. 10.1016/j.chiabu.2021.105372 34717153

[bibr23-13591045251383632] Haycock I. (2023). Is the mental health system in Aotearoa New Zealand providing quality care to young people? A critical analysis utilising the perspectives of mental health professionals and sociological insights. The University of Waikato. https://hdl.handle.net/10289/16466

[bibr24-13591045251383632] Healthify He Puna Waiora . (2024). Mental health services for young people. https://healthify.nz/hauora-wellbeing/m/mental-health-services-for-young-people/

[bibr25-13591045251383632] Hom M. A. Stanley I. H. Rogers M. L. Gallyer A. J. Dougherty S. P. Davis L. Joiner T. E. (2018). Investigating the iatrogenic effects of repeated suicidal ideation screening on suicidal and depression symptoms: A staggered sequential study. Journal of Affective Disorders, 232, 139–142. 10.1016/j.jad.2018.02.022 29486340

[bibr26-13591045251383632] Idenfors H. Kullgren G. Renberg E. S. (2015). Professional care as an option prior to self-harm. Crisis. 10.1027/0227-5910/a000310 26088828

[bibr27-13591045251383632] Jones J. D. Cassidy J. Shaver P. R. (2015). Parents’ self-reported attachment styles: A review of links with parenting behaviors, emotions, and cognitions. Personality and Social Psychology Review: An Official Journal of the Society for Personality and Social Psychology, Inc, 19(1), 44–76. 10.1177/1088868314541858 25024278 PMC4281491

[bibr28-13591045251383632] Kisely S. Yu D. Maehashi S. Siskind D. (2021). A systematic review and meta-analysis of predictors and outcomes of community treatment orders in Australia and New Zealand. The Australian and New Zealand journal of psychiatry, 55(7), 650–665. 10.1177/0004867420954286 32921145

[bibr29-13591045251383632] Lacey C. Huria T. Beckert L. Gilles M. Pitama S. (2011). The Hui process: A framework to enhance the doctor-patient relationship with Māori. The New Zealand medical journal, 124(1347), 72–78. 22237570

[bibr30-13591045251383632] Leddie G. Fox C. Simmonds S. (2022). Nurses’ experiences of working in the community with adolescents who self-harm: A qualitative exploration. Journal of Psychiatric and Mental Health Nursing, 29(5), 744–754. 10.1111/jpm.12806 34797016

[bibr31-13591045251383632] MacDonald S. Sampson C. Biddle L. Kwak S. Y. Scourfield J. Evans R. (2021). Theorising health professionals’ prevention and management practices with children and young people experiencing self-harm: A qualitative hospital-based case study. Sociology of Health & Illness, 43(1), 201–219. 10.1111/1467-9566.13211 33113234 PMC9904420

[bibr32-13591045251383632] Marzetti H. McDaid L. O'Connor R. (2023). A qualitative study of young people's lived experiences of suicide and self-harm: Intentionality, rationality and authenticity. Child and Adolescent Mental Health, 28(4), 504–511. 10.1111/camh.12641 36811313

[bibr33-13591045251383632] McCutcheon L. K. Chanen A. M. Fraser R. J. Drew L. Brewer W. (2007). Tips and techniques for engaging and managing the reluctant, resistant or hostile young person. The Medical Journal of Australia, 187(S7), S64–S67. 10.5694/j.1326-5377.2007.tb01341.x 17908031

[bibr34-13591045251383632] McGorry P. Bates T. Birchwood M. (2018). Designing youth mental health services for the 21st century: Examples from Australia, Ireland and the UK. The British Journal of Psychiatry. Supplement, 202(s54), s30–s35. 10.1192/bjp.bp.112.119214 23288499

[bibr35-13591045251383632] Menzies R. Gluckman P. Poulton R. (2020). Youth mental health in Aotearoa New Zealand: Greater urgency required.

[bibr36-13591045251383632] Miller E. James A. (2025). Child and adolescent mental health services in Aotearoa New Zealand. BJPsych International, 22(2), 32–34. 10.1192/bji.2025.5 40469453 PMC12131043

[bibr37-13591045251383632] Morris A. S. Criss M. M. Silk J. S. Houltberg B. J. (2017). The impact of parenting on emotion regulation during childhood and adolescence. Child Development Perspectives, 11(4), 233–238. 10.1111/cdep.12238

[bibr38-13591045251383632] Moselli M. Casini M. P. Frattini C. Williams R. (2023). Suicidality and personality pathology in adolescence: A systematic review. Child Psychiatry and Human Development, 54(2), 290–311. 10.1007/s10578-021-01239-x 34524583 PMC9977705

[bibr39-13591045251383632] Murray B. L. Wright K. (2006). Integration of a suicide risk assessment and intervention approach: The perspective of youth. Journal of Psychiatric and Mental Health Nursing, 13(2), 157–164. 10.1111/j.1365-2850.2006.00929.x 16608470

[bibr40-13591045251383632] NZ Government . (2018). He Ara Oranga: Report of the government inquiry into mental health and addiction. https://mentalhealth.inquiry.govt.nz/inquiry-report/he-ara-oranga/10.1177/0004867419872810 31672046

[bibr41-13591045251383632] O'Reilly M. Vostanis P. Taylor H. Day C. Street C. Wolpert M. (2013). Service user perspectives of multiagency working: A qualitative study with children with educational and mental health difficulties and their parents. Child and Adolescent Mental Health, 18(4), 202–209. 10.1111/j.1475-3588.2012.00674.x 32847304

[bibr62-13591045251383632] Pearse E. Bucci S. Raphael J. Berry K. (2020). The relationship between attachment and functioning for people with serious mental illness: a systematic review. Nordic Journal of Psychiatry, 74(8), 545–557. 10.1080/08039488.2020.1767687 32692588

[bibr42-13591045251383632] Piao J. Huang Y. Han C. Li Y. Xu Y. Liu Y. He X. (2022). Alarming changes in the global burden of mental disorders in children and adolescents from 1990 to 2019: A systematic analysis for the global burden of disease study. European Child & Adolescent Psychiatry, 31(11), 1827–1845. 10.1007/s00787-022-02040-4 35831670

[bibr43-13591045251383632] Pitama S. Robertson P. Cram F. Gillies M. Huria T. Dallas-Katoa W. (2007). Meihana model: A clinical assessment framework. New Zealand Journal of Psychology, 36(3), 118–125. https://www.psychology.org.nz/journal-archive/Pitamaetal_NZJP36-3_pg118.pdf

[bibr44-13591045251383632] Sadath A. Kavalidou K. McMahon E. Malone K. McLoughlin A. (2024). Associations between humiliation, shame, self-harm and suicidality among adolescents and young adults: A systematic review. PLoS One, 19(2), Article e0292691. 10.1371/journal.pone.0292691 38329967 PMC10852296

[bibr45-13591045251383632] Schienle A. Schwab D. Höfler C. Freudenthaler H. H. (2020). Self-disgust and its relationship with lifetime suicidal ideation and behavior. Crisis. 10.1027/0227-5910/a000645 31918583

[bibr46-13591045251383632] Simes D. Shochet I. Murray K. Sands I. G. (2022). A systematic review of qualitative research of the experiences of young people and their caregivers affected by suicidality and self-harm: Implications for family-based treatment. Adolescent Research Review, 7(2), 211–233. 10.1007/s40894-021-00164-3

[bibr47-13591045251383632] Sroufe L. A. Carlson E. A. Levy A. K. Egeland B. (1999). Implications of attachment theory for developmental psychopathology. Development and Psychopathology, 11, 1–13. 10.1017/s0954579499001923 10208353

[bibr48-13591045251383632] Stace A. (2024). Youth experiences of using a crisis text line. University of Canterbury. https://hdl.handle.net/10092/107078

[bibr49-13591045251383632] Steggals P. Lawler S. Graham R. (2020). The social life of self-injury: Exploring the communicative dimension of a very personal practice. Sociology of Health & Illness, 42(1), 157–170. 10.1111/1467-9566.12994 31552687 PMC7004175

[bibr50-13591045251383632] Stubbing J. Gibson K. (2021). Can we build ‘somewhere that you want to go’? Conducting collaborative mental health service design with New Zealand’s young people. International Journal of Environmental Research and Public Health, 18(19), 9983. 10.3390/ijerph18199983 34639289 PMC8507894

[bibr51-13591045251383632] Stubbing J. Gibson K. (2022). What young people want from clinicians: Youth-informed clinical practice in mental health care. Youth, 2(4), 538-555. 10.3390/youth2040039

[bibr52-13591045251383632] Sutcliffe K. Ball J. Clark T. C. Archer D. Peiris-John R. Crengle S. Fleming T. (2022). Rapid and unequal decline in adolescent mental health and well-being 2012–2019: Findings from New Zealand cross-sectional surveys. The Australian and New Zealand Journal of Psychiatry, 57(2), 264–282. 10.1177/00048674221138503 36453262 PMC10829428

[bibr53-13591045251383632] Sutcliffe K. Wilson M. Clark T. C. Crengle S. Fleming T. (2024). Distinct profiles of mental health need and high need overall among New Zealand adolescents – cluster analysis of population survey data. The Australian and New Zealand Journal of Psychiatry, 58(8), 678–692. 10.1177/00048674241243262 38600641 PMC11308291

[bibr54-13591045251383632] Swee G. Shochet I. Cockshaw W. Hides L. (2020). Emotion regulation as a risk factor for suicide ideation among adolescents and young adults: The mediating role of belongingness. Journal of Youth and Adolescence, 49(11), 2265–2274. 10.1007/s10964-020-01301-2 32772329

[bibr55-13591045251383632] Taylor T. L. Hawton K. Fortune S. Kapur N. (2018). Attitudes towards clinical services among people who self-harm: Systematic review. The British Journal of Psychiatry: The Journal of Mental Science, 194(2), 104–110. 10.1192/bjp.bp.107.046425 19182168

[bibr56-13591045251383632] Telfar S. McLeod G. F. H. Dhakal B. Henderson J. Tanveer S. Broad H. E. T. Woolhouse W. Macfarlane S. Boden J. M. (2023). Child abuse and neglect and mental health outcomes in adulthood by ethnicity: Findings from a 40-year longitudinal study in New Zealand/Aotearoa. Child Abuse & Neglect, 145, Article 106444. 10.1016/j.chiabu.2023.106444 37703676

[bibr57-13591045251383632] Thabrew H. Gandeza E. Bahr G. Bettany D. Bampton C. Cooney E. Coleman N. Tiatia-Seath J. (2018). The management of young people who self-harm by New Zealand infant, child and adolescent mental health services: Cutting-edge or cutting corners? Australasian Psychiatry: Bulletin of Royal Australian and New Zealand College of Psychiatrists, 26(2), 152–159. 10.1177/1039856217748248 29357671

[bibr58-13591045251383632] Wasson Simpson K. S. Gallagher A. Ronis S. T. Miller D. A. A. Tilleczek K. C. (2022). Youths’ perceived impact of invalidation and validation on their mental health treatment journeys. Administration and Policy in Mental Health, 49(3), 476–489. 10.1007/s10488-021-01177-9 34812964

[bibr59-13591045251383632] Williams L. (1998). Personal accounts: A “classic” case of borderline personality disorder. Psychiatric Services, 49(2), 173–174. 10.1176/ps.49.2.173 9574999

[bibr60-13591045251383632] Young W. Joyce L. R. Frampton C. Mulder R. (2025). Adolescent mental health presentations to a New Zealand emergency department: A 16-year retrospective observational study. Australasian Psychiatry: Bulletin of Royal Australian and New Zealand College of Psychiatrists, 33(3), 440–447. 10.1177/10398562251325414 40078119

[bibr61-13591045251383632] Zortea T. C. Gray C. M. O’Connor R. C. (2021). The relationship between adult attachment and suicidal thoughts and behaviors: A systematic review. Archives of Suicide Research: Official Journal of the International Academy for Suicide Research, 25(1), 38–73. 10.1080/13811118.2019.1661893 31545148

